# Annual Report of the Royal Imperial Veterinary School, Vienna, Austria, 1883–84

**Published:** 1885-10

**Authors:** 


### ANNUAL REPORT OF THE ROYAL IMPERIAL VETERINARY SCHOOL, VIENNA, AUSTRIA, 1883-4.

For the regular three years' course there were .........203 students.
For the different grades of horse-shoers during the year.578 "
The diploma of graduation was given to................ 46 "
The library contains 4191 works of 10,774 volumes.
Whole number of animals treated in the Internal and Surgical Clinics............................................3057
Number of Dogs treated in the hospital.......................1347
Number of Dogs examined as rabid............................ 161
Number of Dogs examined as suspicious of rabies.............1142
Number of Dogs examined necroscopically..................... 54
Number of Dogs killed at owner's request....................1198
Number of Horses subjected to a forensic examination for soundness............................................ 215
Number of Horses shod at school forge.......................7876

The report is replete in numerous clinical histories with reference to interesting cases that occurred in the hospital during the year.

